# Medication-related knowledge, administration practice and adherence among caregivers of chronically ill children in Malaysia

**DOI:** 10.1186/s12887-021-02691-3

**Published:** 2021-05-03

**Authors:** Chii-chii Chew, Huan-keat Chan, Chee-tao Chang, Amar-singh HSS, Mohamed azmi Hassali

**Affiliations:** 1 Clinical Research Centre, Hospital Raja Permaisuri Bainun, Ministry of Health, Level 4, Ambulatory Care Centre (ACC), Jalan Raja Ashman Shah, 30450 Ipoh, Perak Malaysia; 2 Social and Administrative Pharmacy, School of Pharmaceutical Sciences, Universiti Sains Malaysia, 11800 Penang, Malaysia; 3 Clinical Research Centre, Hospital Sultanah Bahiyah, Ministry of Health, 05460 Alor Setar, Kedah, Malaysia

**Keywords:** Child, Chronic disease, Caregivers, Parents, Administration and dosage, Medication adherence

## Abstract

**Background:**

Caregivers’ knowledge, practice and adherence in medication administration who care for children with chronic illness requiring long-term pharmacological treatments are factors associating with children medication safety at home. This study aimed to determine the medication-related knowledge, administration practice and adherence among caregivers of chronically ill children in Malaysia. This cross-sectional study was conducted at the paediatric outpatient clinic of a tertiary public hospital. Caregivers of chronically ill children, who engaged in medication administration at home for at least 3 months, were conveniently recruited. Their medication-related knowledge and administration practice were evaluated based on a checklist, while their adherence to medication administration was assessed using a validated 5-point scale. The associated factors were also explored.

**Results:**

Of the 141 participants, most were mothers (90.8%) and had a full-time job (55.3%). Most of them had adequate medication-related knowledge (71.6%) and an appropriate administration practice (83.0%). The majority of them (83.0%) also rated themselves as adherent to medication administration. The participants with a child above 5 years of age (91.2%) were found to have a better practice than those with younger children (75.3%) in medication administration (*p* = 0.012). However, those with a child taking two (adjusted OR: 12.53) or three (adjusted OR: 8.29) medications, getting their refills from private health institutions apart from this hospital (adjusted OR = 7.06) and having multiple illnesses (adjusted OR = 21.25) were more likely to be not adherent to medication administration.

**Conclusion:**

Caregivers of chronically ill children in Malaysia generally have sufficient knowledge and an appropriate practice of medication administration at home. Yet, strategies to improve the adherence to medication administration, particularly in those who care for children with complicated health conditions, are warranted.

## Key messages

1. 71.6% of caregiver had adequate medication-related knowledge
2. 83% of them able to demonstrate appropriate practice in medication administration.
3. 83.0% of the caregivers rated themselves as adherent to medication administration.
4. Non-adherents were more likely among caregivers deal with a complex regime, refill the medications concurrently from private health institutions and the health facilities where the child had been following up, and child have multiple illnesses.

## Introduction

In general, chronic illnesses in childhood refer to diseases taking place in children below 18 years of age, which last longer than 3 months [1]. The global prevalence of chronic illnesses in childhood has grown by 4 times over the past half century [2]. Consequently, the number of children receiving long-term pharmacological treatment is rising across the world [3]. Unlike adults, young patients are heavily dependent on caregivers in taking medications at home [4–6].

However, insufficient knowledge of medications in caregivers has been resulting in countless adverse drug events in children include overdosing, poisoning or increased risk of growth retardation [5]. Their difficulty in reading drug labels, poor understanding of medication instructions, confusion about different brand names of drugs, unfamiliarity with the devices to be used for medication administration and poor communication with their partners in child care are all known factors contributing to medication errors in children [5, 7, 8]. Thus, education about the medications in caregivers of young children is essential [6, 7]. 

While liquid formulation remains the most common form of dosage form for young children, caregivers, especially those from the developing countries, have difficulties in liquid measurement [5, 7, 9]. Notably, dosing errors in children is three times more prevalent than in adults, and such errors could cause harm in children, particularly those who receive long-term treatment [4, 5, 10]. Ideally, caregivers should also maintain a record of their medication administration activities and ensure an effective communication with their partners with whom they share their responsibility [8, 9, 11]. Besides helping to achieve the desired outcomes of the treatment, an appropriate medication administration practice is important to avert medication errors and unnecessary hospital visits [5].

When it comes to ensuring the efficacy of pharmacological treatment in children, another key component is the adherence of the caregivers to medication administration. However, the variations in the degree of parental adherence to medication administration across countries are evident, ranged from 43 to 100% [12]. In fact, non-adherence to medication administration has been reported to have led to suboptimal treatment outcomes [5], and consequently posed a financial burden to the global healthcare system by resulting in an additional annual expenditure per patient between $669 to $162,699 US dollars from year 1997 to 2017 [13].

Good parental knowledge in medication were reported associated with those residing in the Western country and having high socioeconomic status [14]. High educational level in caregivers was one of the significant factors associated with good knowledge in the management of childhood asthma [15]. Higher mothers’ educational levels and household incomes were also factors positively related to adherence in medication administrations [16]. Consistently, caregivers with low educational levels, being single mothers, having busy schedules and other family responsibilities reported more missed doses [8]. On the other hand, parents’ age, education levels, cooking experiences and previous drug administering experiences were not associated with better practice of medication administration, particular in measuring paediatric oral liquid formulation [9]. Another study, however, reported that caregivers’ educational levels were not associated with the practice of medication administration, in particular the selection of dosing instrument [17].

As recently highlighted by the Ministry of Health in Malaysia, studies on how medications for children have been handled outside hospital settings remains limited [18]. This study was designed to determine the medication-related knowledge, administration practice, and adherence among caregivers of chronically ill children in Malaysia. What were the level of knowledge, administration practice and level of adherence among the caregivers with regard to their children’s medications?

## Methods

This cross-sectional study was conducted between January and April 2019 at the paediatric outpatient clinic of a tertiary public hospital located in northern Malaysia. The diseases treated by the clinic ranges from neurological disorders, developmental delay, asthma, kidney diseases to congenital cardiac defects. It has been visited by 30 to 60 patients each day on an appointment basis.

The main caregivers of children under 12 years of age, who had engaged in the medication administration for at least 3 months, were included in the study [1]. Only the mother was selected if both the parents of a child shared the responsibility for the medication administration, as mothers were shown to have played a more prominent role in child care [19]. On the other hand, caregivers who were not able to communicate in the Malay language or English, or whose children had HIV infection, cancer or intellectual impairment, were excluded from the study. The caregivers of these population were not selected mainly because HIV and cancer patients are closely monitored by the healthcare providers and there is a designated pharmacist who will recruit caregivers of HIV patients to attend Medication Therapy Adherence Clinic (MTAC) each time when they come for follow-up. The practices of medication administration among caregivers of children with intellectual impairment are deemed different from that of the general population and hence they were excluded from this study [20].

The participants of the study were recruited using the convenience sampling method. The eligible caregivers were identified from the medical records of their children and were first approached through a telephone call. Written consent was obtained from those who agreed to participate in this study on the day of their following visits to the hospital.

The minimal number of respondents required in this study was 141, calculated by using the formula for prevalence studies with a known population size. It was based on an estimated medication error rate of 33% and an estimated number of eligible patients fulling the inclusion and exclusion criteria presenting to the clinic in a month (*N* = 235) [8]. The confidence level and precision were fixed at 95 and 5%, respectively.

A data collection form adapted from Walsh et al. (2008) was used to collect information regarding i) the characteristics of the caregiver (age, relationship with the child, education level, occupation, and marital status) and ii) the characteristics of the child (age, gender and type of chronic illness), iii) the medication-related information documented in the medical records of patients. The knowledge about the medications (names, indications, times of administration, devices used, treatment durations and the dates of the last doses) and the administration practice (maintaining a medication administration record, communication with partners, measurement accuracy, medication preparation method, administration technique and adherence to the prescribed treatment duration and doses) of caregivers were subsequently assessed by the investigator (pharmacist) based on a checklist. In the cases involving liquid medications, the caregivers were also required to demonstrate how they would measure liquid at home by using oral syringes of different sizes (1 mL, 5 mL and 10 mL) or a measuring cup. A metered-dose inhaler and a spacer were also prepared for the caregivers to demonstrate how these devices should be used at home. The device usage and technique were assessed based on standardised medication counselling guidelines published by the Ministry of Health [21]. The 5-point Medication Adherence Report Scale (MAR-5) was used to allow caregivers to rate their adherence level in the medication administration over the past one month. The questionnaire (MARS-5) adapted in both English and Malay languages was validated for its construct validity and reliability (internal consistency and test-retest reliability), and it demonstrated acceptable psychometric properties [22]; in the context of publication right of MARS, the psychometric data are not allowed to be included in this article as per the agreement between the author and the originator. Both the checklist and questionnaire were pre-tested for understanding prior to data collection. Ethical approval from Medical Research Ethics Committee, Ministry of Health Malaysia was obtained [Research registration number: NMRR-18-228-40,047 (IIR)] and written informed consent from the participants was sought prior to data collection.

The participants were considered to have the right knowledge about the medications if their responses were consistent with the documentation in the medical records of their children and able to provide the details of the medications correctly based on the checklist. Participants were allowed to give either commercial or generic name when they were asked about the medication name. Their practice in medication administration was deemed to be appropriate only if they communicated with their partners or other family members immediately following the medication administration, demonstrated accurate liquid measurement and device handling techniques, and administered the medications at doses and for duration as prescribed.

The adherence level of a caregiver in the medication administration was indicated by a mean score with a possible range from 6 to 30, and a score below 27 indicated non-adherence [23]. The factors associated with the poor knowledge, inappropriate practice and poor adherence, as well as their interactions, were explored using the Pearson’s chi-square analysis [24]. The stepwise backward logistic regression analysis was also performed on all the variables with a *p*-value below 0.05 to identify the predictors of poor adherence. The final model was tested for interactions and multicollinearity between the variables, and its fitness was tested using the Hosmer-Lemeshow goodness-of-fit test.

## Results

Of the 141 participants enrolled in this study, most were mothers (90.8%) and married (95.7%). They had a mean age of 35.8 (SD: 6.0) years. The majority of them had received at least secondary school education (95.8%) and had a full-time job (55.3%). Nearly 60% of caregivers received helps in the medication administration, mainly from their spouses. On average, they had engaged in the medication administration for 26 (IQR: 26) months. Almost all of them (95.1%) were able to read the label without help from the others. Most of them (58.9%) dealt with at least two types of medications. Their children were aged 5.45 (SD: 3.39) years on average and mostly had only one type of disease (97.9%) (Table 1).

**Table 1:** Characteristic of caregivers and children

Demographic characteristics	n (%); *n* = 141
**Caregivers**	
Relationship with child	
Mother	128 (90.8)
Father	12 (8.5)
Relative	1 (0.7)
Receiving help from	
Another parent	55 (39.0)
Other family members	27 (19.1)
None	59 (41.8)
Highest educational level	
Secondary education	83 (58.9)
Tertiary education	52 (36.9)
No formal education/ Primary education	6 (4.2)
Occupation	
Stay-home parents	63 (44.7)
Professional	19 (13.5)
Semi-professional	45 (31.9)
Labourer	14 (9.9)
Marital status	
Married	135 (95.7)
Single	6 (4.3)
Requiring help to read medication labels?	
Never	134 (95.1)
Rarely	3 (2.1)
Always	1 (0.7)
Never reading	3 (2.1)
Sources of medications	
Only this hospital	119 (84.4)
Other government-funded hospital/clinic	7 (5.0)
Private pharmacy/clinic/hospital	10 (7.0)
Number of medications administered to children	
1	58 (41.1)
2	62 (44.0)
3	18 (12.8)
4	3 (2.1)
**Children**	
Age, year [mean (SD)]	5.45 (3.39)
Gender	
Male	78 (55.3)
Female	63 (44.7)
Number of chronic illnesses	
1	138 (97.9)
≥ 2	3 (2.1)
**Pharmaceutical Dosage Form (*n*** **= 248)**^a^	
Inhalation	110 (44.4)
Oral liquid	65 (26.2)
Tablet	54 (21.8)
Dispersible tablet	14 (5.6)
Nasal spray	2 (0.8)
Intravenous injection	1 (0.4)
Granules	1 (0.4)
Gargle solution	1 (0.4)

^a^there was a total of 248 medication being assessed

Most participants (71.6%) were found to have sufficient overall knowledge of the medications. They generally had the right knowledge of the indications (82.3%), the prescribed time and frequency (92.2%), the prescribed dosage (92.9%), and the devices to be used (100%) for the medications (Table 2).

**Table 2:** Medication-related knowledge, administration practice and adherence of caregivers

Variables	Correct /Appropriate n (%); n = 141
Knowing the names of medications	66 (46.8)
Maintaining a record for medication administration	61 (43.3)
**Overall medication-related knowledge**	
**Adequate**	**104 (71.6)**
**Inadequate**	**40 (28.4)**
**Individual aspect of medication-related knowledge**	
Knowing the indications	116 (82.3)
Knowing the time and frequency of administration	130 (92.2)
Knowing the device for administration	119 (100)
Not involving a device	22
Knowing the dosage	131 (92.9)
**Overall practices of medication administration**	
**Appropriate**	**117 (83.0)**
**Inappropriate**	**24 (17.0)**
Always communicating with partners (*n* = 82)	72 (87.8)
Accurate dosage measurement for oral liquid medication (*n* = 49)	43 (87.8)
Appropriate method of medication preparation	137 (97.2)
Appropriate technique of administration	139 (98.6)
Appropriate date of last dose administration	133 (94.3)
Appropriate dosage	139 (98.6)
**Medication adherence report scale (MARS)**	
**Not adherent (< 27 Scores)**	**24 (17.0)**
**Adherent**	**117 (83.0)**

Most of them (83.0%) had an appropriate practice in the medication administration. In general, they did communicate with their helpers immediately after the medication administration (87.8%), performed the liquid measurement correctly (87.8%), and demonstrated the correct medication preparation (97.2%) and administrations (98.6%) techniques. A small number of them (5.7%) discontinued the treatment of their children prematurely without seeking advices from physicians (Table 2). An inappropriate medication administration practice was more commonly seen in the participants with a child under 5 years of age (*p* = 0.012) (Table 3).

**Table 3:** The associations between characteristics of caregivers and their knowledge of, practice of, and adherence to medication administration

Variables	Knowledge			Practice			Adherence		
	Adequate *n* = 101	Inadequate *n* = 40	P-value^a^	Appropriate *n* = 117	Inappropriate n = 24	P-value^a^	Adherence n = 117	Non-adherent n = 24	P-value^a^
Caregivers mean age (SD)	36.32 (5.90)	34.58 (6.05)	0.119^b^	35.93 (6.05)	35.29 (5.63)	0.634^b^	35.85 (5.96)	35.71 (6.14)	0.918^b^
Relationship with the child			0.515			0.744			0.353
Mother	93 (72.7)	35 (27.3)		105 (82.0)	23 (18.0)		104 (81.2)	24 (18.8)	
Father	7 (58.3)	5 (41.7)		11 (91.7)	1 (8.3)		12 (100.0)	0 (0.0)	
Relatives	1 (100.0)	0 (0.0)		1 (100.0)	0 (0.0)		1 (100.0)	0 (0.0)	
Receiving help from			0.861			0.296			0.660
Another parent	38 (69.1)	17 (30.9)		49 (89.1)	6 (10.9)		45 (81.8)	10 (18.2)	
Other family members	20 (74.1)	7 (25.9)		21 (77.8)	6 (22.2)		24 (88.9)	3 (11.1)	
None	43 (72.9)	16 (27.1)		47 (79.7)	12 (20.3)		48 (81.4)	11 (18.6)	
Highest educational level			0.100			0.139			0.571
No formal / Primary education	2 (33.3)	4 (66.7)		5 (83.3)	1 (16.7)		5 (83.3)	1 (16.7)	
Secondary education	62 (74.4)	21 (25.3)		73 (88.0)	10 (12.0)		71 (85.5)	12 (14.5)	
Tertiary education	37 (71.2)	15 (28.8)		39 (75.0)	13 (25.0)		41 (78.8)	11 (21.2)	
Occupation			0.067			0.093			0.220
Stay-home parents	50 (79.4)	13 (20.6)		56 (88.9)	7 (11.1)		55 (87.3)	8 (12.7)	
Working caregiver	51 (65.4)	27 (34.6)		61 (78.2)	17 (21.8)		62 (79.5)	16 (20.5)	
Marital status			1.000			0.270			0.270
Married	97 (71.9)	38 (28.1)		113 (83.7)	22 (16.3)		113 (83.7)	22 (16.3)	
Single	4 (66.7)	2 (33.3)		4 (66.7)	2 (33.3)		4 (66.7)	2 (33.3)	
Duration in medications administration, months			0.829			0.074			0.788
≤ 12	26 (68.4)	12 (31.6)		28 (73.7)	10 (26.3)		31 (81.6)	7 (18.4)	
> 12	75 (72.8)	28 (27.2)		89 (86.4)	14 (13.6)		86 (83.5)	17 (16.5)	
Requiring help to read medication labels?			0.539			1.000			1.000
Never	97 (72.4)	37 (27.6)		110 (82.1)	24 (17.9)		110 (82.1)	24 (17.9)	
Rarely	2 (66.7)	1 (33.3)		3 (100.0)	0 (0.0)		3 (100.0)	0 (0.0)	
Always	0 (0.0)	1 (100.0)		1 (100.0)	0 (0.0)		1 (100.0)	0 (0.0)	
Never reading	2 (66.7)	1 (33.3)		3 (100.0)	0 (0.0)		3 (100.0)	0 (0.0)	
Where else do you get your medications?			0.928			0.461			**0.020**
Only this hospital	84 (70.6)	35 (29.4)		99 (83.2)	20 (16.8)		102 (85.7)	17 (14.3)	
Other government funded hospital/clinic	9 (75.0)	3 (25.0)		11 (91.7)	1 (8.3)		10 (83.3)	2 (16.7)	
Private pharmacy/clinic/ hospital	8 (80.0)	2 (20.0)		7 (70.0)	3 (30.0)		5 (50.0)	5 (50.0)	
Number of medications administered			0.863			0.191			**< 0.001**
1	42 (72.4)	16 (27.6)		51 (87.9)	7 (12.1)		56 (96.6)	2 (3.4)	
≥ 2	59 (71.1)	24 (28.9)		66 (79.5)	17 (20.5)		61 (73.5)	22 (26.5)	
Children age group, years			0.791			**0.012**			0.480
≤ 5	53 (72.6)	20 (27.4)		55 (75.3)	18 (24.7)		59 (80.8)	14 (19.2)	
> 5	48 (70.6)	20 (29.4)		62 (91.2)	6 (8.8)		58 (85.3)	10 (14.7)	
Children gender			0.962			0.744			0.054
Male	56 (71.8)	22 (28.2)		64 (82.1)	14 (17.9)		69 (88.5)	9 (11.5)	
Female	45 (71.4)	18 (28.6)		53 (84.1)	10 (15.9)		48 (76.2)	15 (23.8)	
Number of Chronic diseases			1.000			1.000			0.075
One type	99 (71.1)	39 (28.3)		114 (82.6)	24 (17.4)		116 (84.1)	22 (15.9)	
Two types	2 (66.7)	1 (33.3)		3 (100.0)	0 (0.0)		1 (33.3)	2 (66.7)	

^a^Analysed with Pearson Chi-square analysis. Fisher’s exact test was applied when assumptions for Pearson Chi-square test was not met

^b^ analysed with independent T-test

More than 80% of the caregivers rated themselves as adherent to the medication administration (Table 2). The type of health institutions from which they obtained their refills besides this hospital (*p* = 0.020) and the number of medications taken by the child (*p* < 0.001) were significantly associated with their adherence level (Table 3). The caregivers with children taking a greater number of medications, obtaining refills from private health institutions and with more than one chronic illness had higher odds of being non-adherent to the medication administration (Table 4).

**Table 4:** Predictors for non-adherence to medication administration, multiple logistic regression analysis

Factors associated with not adherents	Not-adherents	
	Adj. OR (95% CI)	p-value
Number of medications administered to children		
1	1.00	
2	**12.53 (2.47, 63.33)**	0.002*
3	**8.29 (1.21, 56.62)**	0.031*
4	9.83 (0.01, 219.01)	0.149
Sources of medications		
Only this hospital	1.00	
Other government funded hospital/clinic	1.04 (0.19, 5.55)	0.968
Private pharmacy/clinic/hospital	**7.06 (1.49, 33.45)**	0.014*
Number of Chronic diseases of children		
One type of chronic disease	1.00	
Two types of chronic disease	**21.25 (1.16, 388.43)**	0.039*

Adj. OR: Adjusted odd ratio, CI: Confidence interval, *p-value < 0.05

## Discussion

The findings of this study suggest that most caregivers of young children have adequate knowledge about medications and appropriate practice in their administration. In addition, they also have a high adherence level in the medication administration. However, those with children taking a greater number of medications, getting their refills from private health institutions and with more complicated medical conditions were more likely to be less adherent to the medication administration. The findings also help us better understand the issues related to the medication administration in the context of Malaysia [18], and could be used to guide healthcare providers in educating caregiver.

Consistent with the finding of a previous study [8], this study shows that children under 5 years of age are more likely to be victims of an inappropriate medication administration practice. The absence of age-appropriate drug formulations commonly requires caregivers to crush a tablet, reconstitute a powder formulation and accurately measure a liquid formulation [5]. Inappropriate practices are likely to be unavoidable due to the lack of proper techniques and devices, as well poor palatability of the medications [5, 9, 25]. Apart from relying on the pharmaceutical to develop more child-friendly formulations [5], health care providers could educate caregivers how to improve the acceptance of medications in children include informing the caregiver if the medications were compatible with food and masking the poor taste with certain food or drink (e.g. juice) [26] and adopt several strategies well supported by the existing literature, including the use of pictographic instructions and the teach-back method, to avert inappropriate medication administration practices [27–29].

Caregivers were found to be well informed about their children’s medications in general. This suggests that health care providers have been providing them with sufficient medication-related information, as highly recommended in the existing guidelines [21, 30]. In contrast with caregivers from other developing countries who still tended to use a household spoon for medication administration [7, 9], all participants in this study managed to measure the medication correctly by using a proper measuring device. However, unlike other countries [25, 31], caregivers in Malaysia were commonly not familiarized with the generic names of medications [21, 30]. To avoid errors caused by the confusion, it is important to educate them about different names used for a medication, in addition to its indication, dosage, frequency and treatment duration [27].

Having an appropriate practice in the medication administration in most caregivers in this study was suggestive of the sufficiency and accuracy of the medication instructions they received [21, 30]. Only caregivers who are able to handle the medications correctly at home can shield their children from undesirable adverse events [5]. While dosing errors are common with liquid formulations [7, 8, 32], it is found that most caregivers in this study were able to perform the measurement according to the instructions.

Non-adherence to the medication administration was more common in the caregivers with children taking a greater number of medications, having multiple medical conditions, and getting their refills from private health institutions apart from this hospital. The need to deal with a complicated treatment regimen has long been a known factor for non-adherence among the caregivers of young children [8]. Users who refilled medications from different health institutions may be facing variations in healthcare services, different adherence counselling structure and varied practice of health care providers in providing adherence educations which may result in decreased adherence [12]. In addition, the absence of an effective information exchange mechanism across different health institutions from which caregivers get their refills could lead to repeated administration of the same medication. While the practice of refilling medications from different institutions to overcome logistic hassles may be beneficial [33], further investigation may be required to explore other reasons of such practice.

As this study was limited to its single-centre design, a similar assessment could be conducted on a larger scale. Convenience sampling of urban population may not reflect reality of all caregivers. Also, the assessment was based on self-reporting of caregivers, and therefore the findings might not reflect their actual practice. It would be more ideal if a direct observation on the medication administration practice at home could be performed in future studies [6, 8]. Additionally, knowledge of medication side effect among the caregivers could be evaluated in the future in order to better understand if this is one of the reason of medication non-adherence.

## Conclusion

Most of the caregivers in Malaysia had adequate knowledge about the medications received by their children, as well as an appropriate practice and an acceptable adherence level in the medication administration. However, the medication administration practice in caregivers of children below 5 years of age could be further improved. It is also important to ensure the adherence of caregivers of children with complex medical conditions and taking multiple medications. They also need to be advised to get their refills only at the same health institution from which, whenever possible.

## Data Availability

The datasets generated and/or analysed during the current study are not publicly available due to confidentiality of patients, but are available from the corresponding author on reasonable request.
